# Integrated clinical and genomic models using machine-learning methods to predict the efficacy of paclitaxel-based chemotherapy in patients with advanced gastric cancer

**DOI:** 10.1186/s12885-024-12268-9

**Published:** 2024-04-20

**Authors:** Yonghwa Choi, Jangwoo Lee, Keewon Shin, Ji Won Lee, Ju Won Kim, Soohyeon Lee, Yoon Ji Choi, Kyong Hwa Park, Jwa Hoon Kim

**Affiliations:** 1 Department of Computer Science and Engineering, Korea University, Seoul, Korea; 2 OncoMASTER Inc., Seoul, Korea; 3 Institute of Human Behavior & Genetic, Korea University College of Medicine, Seoul, Korea; 4 Biomedical Research Center, Korea University Anam Hospital, Seoul, Korea; 5 Division of Medical Oncology, Department of Internal Medicine, Korea University College of Medicine, Korea University Anam Hospital, 73, Goryeodae-ro, Seongbuk-gu, Seoul, 02841 Republic of Korea

**Keywords:** Advanced gastric cancer, Paclitaxel, Machine learning, Prediction, Artificial neural network

## Abstract

**Background:**

Paclitaxel is commonly used as a second-line therapy for advanced gastric cancer (AGC). The decision to proceed with second-line chemotherapy and select an appropriate regimen is critical for vulnerable patients with AGC progressing after first-line chemotherapy. However, no predictive biomarkers exist to identify patients with AGC who would benefit from paclitaxel-based chemotherapy.

**Methods:**

This study included 288 patients with AGC receiving second-line paclitaxel-based chemotherapy between 2017 and 2022 as part of the K-MASTER project, a nationwide government-funded precision medicine initiative. The data included clinical (age [young-onset vs. others], sex, histology [intestinal vs. diffuse type], prior trastuzumab use, duration of first-line chemotherapy), and genomic factors (pathogenic or likely pathogenic variants). Data were randomly divided into training and validation sets (0.8:0.2). Four machine learning (ML) methods, namely random forest (RF), logistic regression (LR), artificial neural network (ANN), and ANN with genetic embedding (ANN with GE), were used to develop the prediction model and validated in the validation sets.

**Results:**

The median patient age was 64 years (range 25–91), and 65.6% of those were male. A total of 288 patients were divided into the training (*n* = 230) and validation (*n* = 58) sets. No significant differences existed in baseline characteristics between the training and validation sets. In the training set, the areas under the ROC curves (AUROC) for predicting better progression-free survival (PFS) with paclitaxel-based chemotherapy were 0.499, 0.679, 0.618, and 0.732 in the RF, LR, ANN, and ANN with GE models, respectively. The ANN with the GE model that achieved the highest AUROC recorded accuracy, sensitivity, specificity, and F1-score performance of 0.458, 0.912, 0.724, and 0.579, respectively. In the validation set, the ANN with GE model predicted that paclitaxel-sensitive patients had significantly longer PFS (median PFS 7.59 vs. 2.07 months, *P* = 0.020) and overall survival (OS) (median OS 14.70 vs. 7.50 months, *P* = 0.008). The LR model predicted that paclitaxel-sensitive patients showed a trend for longer PFS (median PFS 6.48 vs. 2.33 months, *P* = 0.078) and OS (median OS 12.20 vs. 8.61 months, *P* = 0.099).

**Conclusions:**

These ML models, integrated with clinical and genomic factors, offer the possibility to help identify patients with AGC who may benefit from paclitaxel chemotherapy.

**Supplementary Information:**

The online version contains supplementary material available at 10.1186/s12885-024-12268-9.

## Background

Over the past decades, fluoropyrimidines (5-fluorouracil, capecitabine, and S-1), platinum (cisplatin and oxaliplatin), taxanes (docetaxel and paclitaxel), and irinotecan have demonstrated survival benefits for the treatment of patients with unresectable or metastatic gastric cancer. Fluoropyrimidine- or platinum-based regimens are widely accepted first-line therapies for patients with advanced gastric cancer (AGC) [1]. Since the REGARD and RAINBOW studies [2, 3], a combination of ramucirumab, a monoclonal antibody targeting vascular endothelial growth factor receptor-2, and paclitaxel has been widely used, and irinotecan has been recommended as a second- or later-line treatment [4, 5]. Recently, novel treatment strategies, including immune checkpoint inhibitors and new targeted inhibitors, have improved the survival of patients with AGC [6–10].

The proportion of patients receiving second- or later-line treatment, along with response and survival rates, have progressively decreased than those observed in first-line treatment [1]. Certain patients may experience clinical deterioration with rapid progression, resulting in missed opportunities for further treatment. Patient fragility, stemming from prior chemotherapy exposure and various disease characteristics, could contribute to this phenomenon. Several factors, such as poor performance status or cumulative toxicity due to the first-line chemotherapy, extent of disease, and history of agents used as the first-line therapy, could influence whether a patient benefits from further treatment [11–14]. Issues have been continuously raised to identify patients who are more likely to benefit from second- or later-line therapy, especially vulnerable patients with AGC.

Through recent next-generation sequencing (NGS), molecular classification of heterogeneous AGC has become more important and its prognostic significance with chemotherapy efficacy is well known [15]. Specifically, taxanes are considered to exhibit anti-cancer effects through aberrant stabilization of microtubules, causing defects in chromosome segregation, mitotic arrest and activation of the spindle assembly checkpoint, where prolonged activation results in cell death. There were previous studies suggesting that altered expression of genes involved in the spindle assembly checkpoint may affect cellular sensitivitiy to paclitaxel [16–18]. However, there are still no definite predictive biomarkers for each palliative chemotherapy in AGC.

Machine learning (ML), a form of artificial intelligence (AI), is widely used and has great potential in precision oncology. Random forest (RF) utilizes multiple decision trees trained on random subsets of data to collectively make predictions for classification or regression tasks. Each tree independently learns the optimal feature splits, and the final prediction is determined by aggregating the outputs from these trees. Logistic regression (LR) is a statistical method used for binary classification that estimates the probability of a binary outcome. It models the relationship between one or more independent variables and a dependent variable using a logistic function, transforming the inputs into probabilities between zero and one. An artificial neural network (ANN) is a computational model consisting of interconnected nodes, called neurons, organized in layers to process information. Through training, ANNs adjust the connections between neurons to learn patterns and make predictions based on the data. Earlier studies have attempted to predict overall survival (OS) and disease-free survival in patients with gastric cancer and the benefits of adjuvant chemotherapy using ML-based methods [18, 19]. Recently, various methods for generating continuously distributed representations of words, for example, Word2Vec [20], have been introduced for joint use with ANN-based machine learning techniques. Similar attempts to represent genetic mutations or protein sequences in a continuous vector space have been made in the biomedical domain [21, 22], showing remarkable improvements in the ability to capture the characteristics of proteins or relationships between mutations.

This study aimed to develop a prediction model to identify patients with AGC who would benefit from paclitaxel-based chemotherapy after failure of fluoropyrimidine and platinum-based chemotherapy.

## Materials and methods

### Patients and K-MASTER datasets

**Fig. 1:**
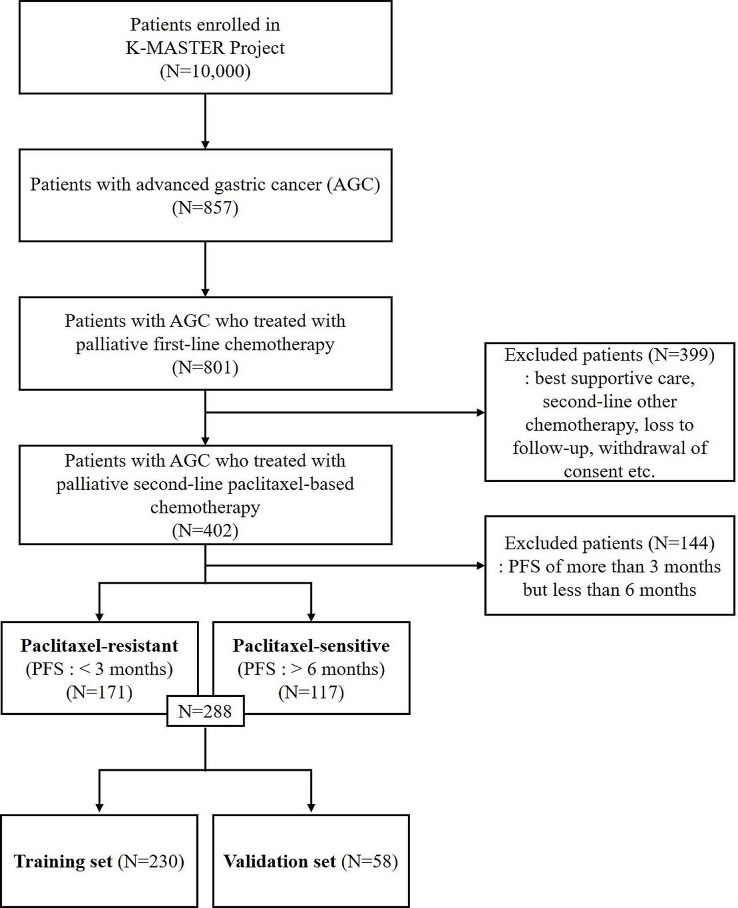
Patient flow diagram

Patients eligible for the study were 20 years of age or older, diagnosed with metastatic or recurrent stomach adenocarcinoma through histological or cytological methods, following the unsuccessful treatment with first-line fluoropyrimidine- and platinum-based chemotherapy. These individuals participated in the second-line, paclitaxel-based chemotherapy as part of the K-MASTER project from 2017 to 2022 (Fig. 1) [23]. The K-MASTER initiative, a comprehensive precision medicine trial across 51 Korean institutions, focused on identifying treatable mutations through Next-Generation Sequencing (NGS) in 10,000 Korean patients with advanced solid tumors, and led to the strategic enrollment of patients in clinical trials tailored to their genetic profiles [23]. Additionally, the K-MASTER involved a nationwide effort to map out genomic profiles and systematically gather data on common clinical attributes across various solid tumors [23].

### Clinical and genetic features

Clinical data included age, sex, tumor histology (intestinal vs. diffuse), prior trastuzumab use, and duration of first-line chemotherapy. The clinical utility of NGS in the K-MASTER project was previously reported [23–25]. The dataset included molecular changes, such as single nucleotide variants, insertions, deletions, copy number variations, and structural variants, all of which have the potential to influence clinical decision-making (Additional file 1). These genetic alterations were classified within databases as either “likely-pathogenic” or “pathogenic” according to COSMIC and ClinVar, and as “likely-oncogenic” or “oncogenic” according to OncoKB databases.

### Preprocess data

Participants in this study were divided into two groups based on their response to second-line paclitaxel-based chemotherapy: those who experienced progression-free survival (PFS) of more than six months were considered paclitaxel-sensitive, while individuals with a PFS of less than three months were deemed paclitaxel-resistant. Following this classification, the cohort was then randomly split into training and validation datasets in an 80:20 ratio, utilizing the ‘StratifiedShuffleSplit’ function from the scikit-learn library to maintain an equal distribution of outcomes across both datasets (Fig. 1).

Clinical and genetic information was subsequently converted into binary form to facilitate analysis, resulting in distinct sets of binary clinical and genetic features. For genetic embeddings, each patient’s genetic variants were first assigned to a vector space, creating vector representations for individual variants. These vectors were aggregated to form a comprehensive genetic feature for each patient. These vector representations were initially set at random values and tuned throughout the training phase.

### Development and validation of ML models in training and validation sets

**Fig. 2:**
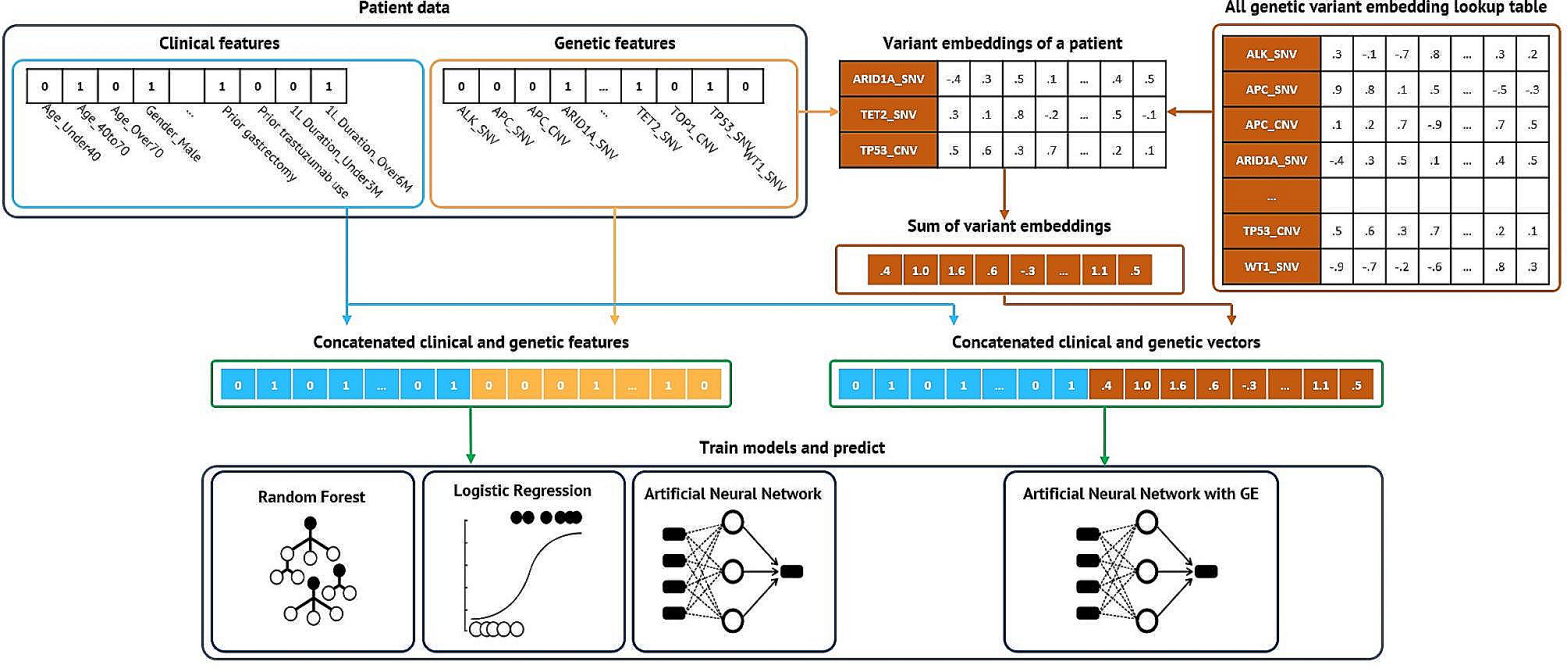
Structures of random forest, logistic regression, artificial neural network, and artificial neural network with genetic embedding

Four ML models – Random Forest (RF), Logistic Regression (LR), Artificial Neural Network (ANN), and ANN incorporating genetic embedding (ANN with GE) – were employed on the training sets and then tested on the validation sets. Patient data was synthesized by combining clinical binary features and genetic information into comprehensive feature vectors. For the LR, RF, and standard ANN models, genetic information was represented through binary genetic features. Conversely, the ANN with GE model utilized vectors of embedded genetic features. These comprehensive patient feature vectors were then used to train each respective model (Fig. 2). The genetic embedding dimension was set at 20, and both ANN configurations included a single hidden layer with 20 nodes. The effectiveness of the models, particularly in predicting patient outcomes following second-line paclitaxel-based chemotherapy, was measured by the area under the Receiver Operating Characteristics (ROC) curve (AUROC), focusing on the progression-free survival (PFS) duration.

Model development and training processes were executed using Python version 3.9.12. The LR, RF, and standard ANN models used tools from the scikit-learn package version 1.1.1, specifically ‘LogisticRegression’, ‘RandomForestClassifier,’ and ‘MLPClassifier’, respectively. The ANN with GE model was developed using functionalities from the PyTorch package, version 1.13.0.

### Statistical analysis

All statistical analyses, along with the development of prediction models, were conducted using Python (version 3.9.12) with the scikit-learn (version 1.1.1) and lifelines (version 0.27.7) packages. PFS was measured from the start of second-line paclitaxel-based chemotherapy until the occurrence of progression or death from any cause. OS was also calculated from the commencement of the same chemotherapy until death due to any cause. Survival rates were determined using the Kaplan-Meier method, and differences between survival curves were assessed with the log-rank test. Statistical significance was established at a *P-*value of less than 0.05, using a two-sided test.

## Results

### Patient characteristics

A total of 288 patients with AGC were treated with second-line paclitaxel-based chemotherapy between 2017 and 2022 (Fig. 1). The median age was 63 years (range 25–91), and 65.6% of patients were male. First-line chemotherapy included fluoropyrimidine- and platinum-based regimes, with trastuzumab use observed in 16.7% of the patients. Second-line chemotherapy comprised paclitaxel + ramucirumab administration to 237 patients (82.3%), while the other patients were treated with paclitaxel alone or paclitaxel + others in clinical trials. The patients were divided into training (*n* = 230) and validation sets (*n* = 58). There were no significant differences in baseline characteristics between the training and validation sets (Table 1). All pathogenic variants observed in all patients were used as genetic features, comprising 73 SNVs and 29 CNVs for 87 genes.

**Table 1:** Baseline characteristics

	Training set (*N* = 230, %)	Validation set (*N* = 58, %)	*P*
Age (y)			
Median, years (range)	64 (25–91)	62 (28–89)	0.154
< 40 years, n (%)	8 (3.5)	5 (8.6)	0.153
≥ 70 years, n (%)	74 (32.2)	14 (24.1)	
Sex			0.209
Male	155 (67.4)	34 (58.6)	
Female	75 (32.6)	24 (41.4)	
Histology			0.146
Intestinal type	143 (62.1)	42 (72.4)	
Diffuse type*	87 (37.8)	16 (27.6)	
Clinical settings			0.835
Initially metastatic disease	154 (67.0)	38 (65.6)	
Recurrent disease	76 (33.0)	20 (34.5)	
Prior gastrectomy	120 (52.2)	26 (44.8)	0.353
Prior trastuzumab use	40 (17.4)	8 (13.8)	0.511
Duration of prior 1st -line fluoropyrimidine plus platinum– based chemotherapy			0.508
< 3 months	57 (24.8)	16 (27.6)	
≥ 6 months	87 (37.8)	19 (32.7)	

^*^ Mixed type was categorized into diffuse type: training set (*n* = 13) and validation set (*n* = 1).

At a median follow-up duration of 19.07 months (95% confidence interval [CI], 15.947–22.193), median PFS and OS were 2.70 months (95% CI, 2.364–3.036) and 13.28 months (95% CI, 10.271–16.289), respectively. Comparisons between the training and validation sets revealed no notable differences in PFS and OS following second-line paclitaxel-based chemotherapy. Specifically, the median PFS was 2.53 months in the training set versus 2.79 months in the validation set (*P* = 0.911), and the median OS was 13.61 months in the training set versus 10.45 months in the validation set (*P* = 0.280).

### Development of four ML-based prediction models

The baseline characteristics of the paclitaxel-sensitive (*n* = 93) and paclitaxel-resistant (*n* = 137) patients in the training set showed no substantial variations (Table 2). The only exception was the length of prior first-line chemotherapy. Specifically, patients with paclitaxel-sensitive advanced gastric cancer (AGC) experienced a significantly longer duration of initial chemotherapy compared to those who were paclitaxel-resistant (47.3% versus 31.4%, *P* = 0.034).

**Table 2:** Baseline characteristics between paclitaxel-sensitive and–resistant patients in the training set

	Paclitaxel-sensitive (*N* = 93, %)	Paclitaxel-resistant (*N* = 137, %)	*P*
Age (y)			
Median, years (range)	64 (37–87)	64 (25–91)	0.910
< 40 years, n (%)	3 (3.2)	5 (3.6)	0.833
$\ge$70 years, n (%)	28 (30.1)	46 (33.6)	
Sex			0.116
Male	57 (61.3)	98 (71.5)	
Female	36 (28.5)	39 (28.5)	
Histology			0.072
Intestinal type	51 (54.8)	92 (67.2)	
Diffuse type	42 (45.2)	45 (32.8)	
Clinical settings			0.132
Initially metastatic disease	57 (61.3)	97 (70.8)	
Recurrent disease	36 (38.7)	40 (29.2)	
Prior gastrectomy	56 (60.2)	64 (46.7)	0.059
Prior trastuzumab use	20 (21.5)	20 (14.6)	0.215
Duration of prior 1st -line fluoropyrimidine plus platinum– based chemotherapy			0.034
< 3 months	17 (18.3)	40 (29.2)	
≥ 6 months	44 (47.3)	43 (31.4)	

**Fig. 3:**
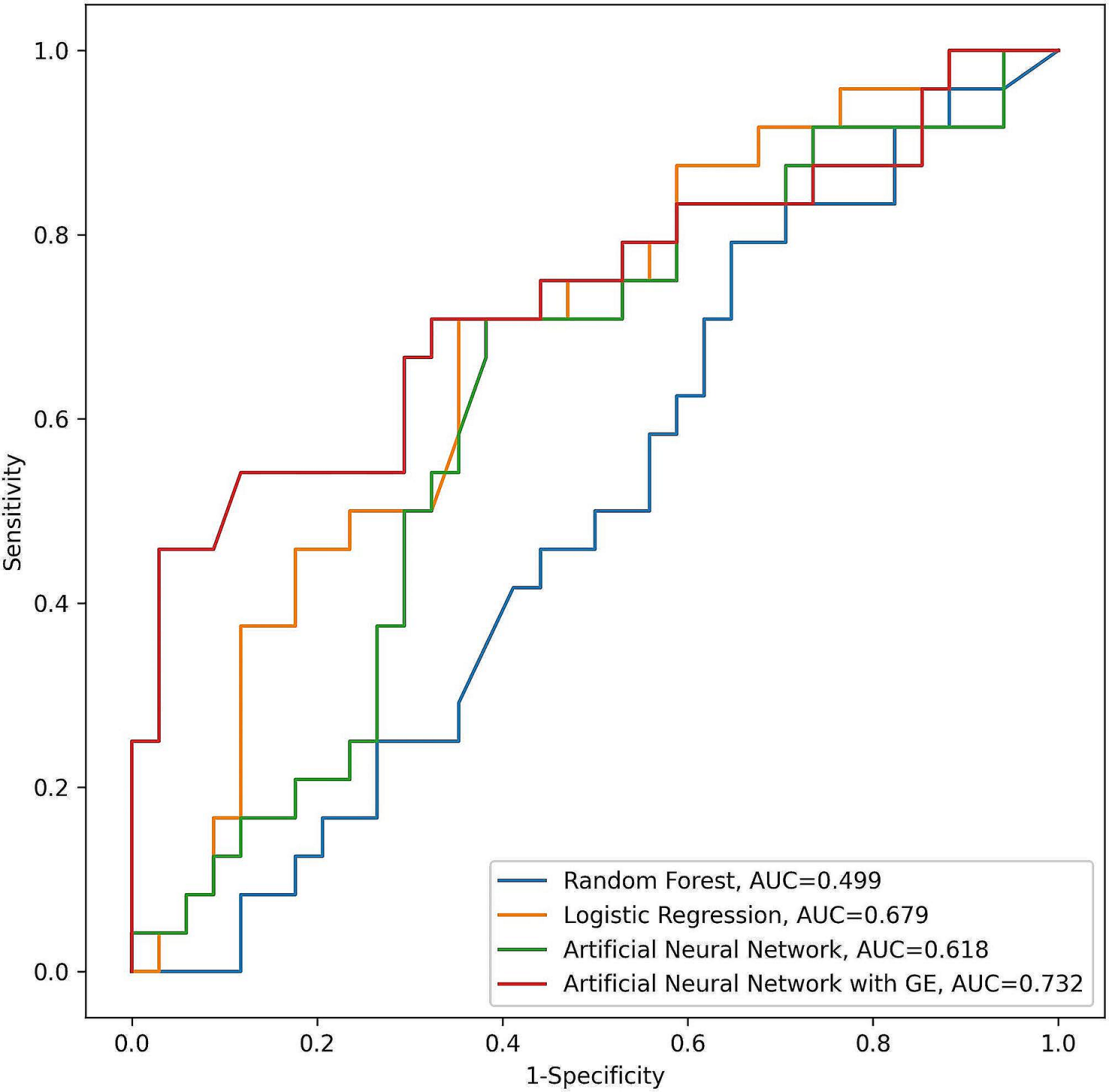
Receiver operating characteristic curves for progression-free survival

The AUROC scores for predicting paclitaxel-sensitive patients varied across different models: 0.499 (95% CI 0.378–0.626) for the RF, 0.679 (95% CI 0.562–0.798) for the LR, 0.597 (95% CI 0.475–0.722) for the ANN, and 0.732 (95% CI 0.610–0.842) for the ANN with E models (Fig. 3). The sensitivity, specificity, accuracy, and F1 scores for these models are detailed in Table 3. Among these, the ANN with GE model demonstrated the highest effectiveness with an AUROC of 0.732, whereas the RF model was the least effective, recording an AUROC of 0.499.

**Table 3:** Performance metrics of machine learning models to predict the progression-free survival of second-line paclitaxel in patients with advanced gastric cancer

Models	AUROC	Sensitivity	Specificity	Accuracy	F1 score
RF	0.499	0.517	0.417	0.588	0.417
LR	0.679	0.638	0.375	0.823	0.461
ANN	0.618	0.500	0.706	0.621	0.522
ANN with GE	0.732	0.458	0.912	0.724	0.579

RF, random forest; LR, logistic regression; ANN, artificial neural network; ANN with GE, artificial neural network with genetic embedding

### Validation of four ML-based prediction models

**Fig. 4:**
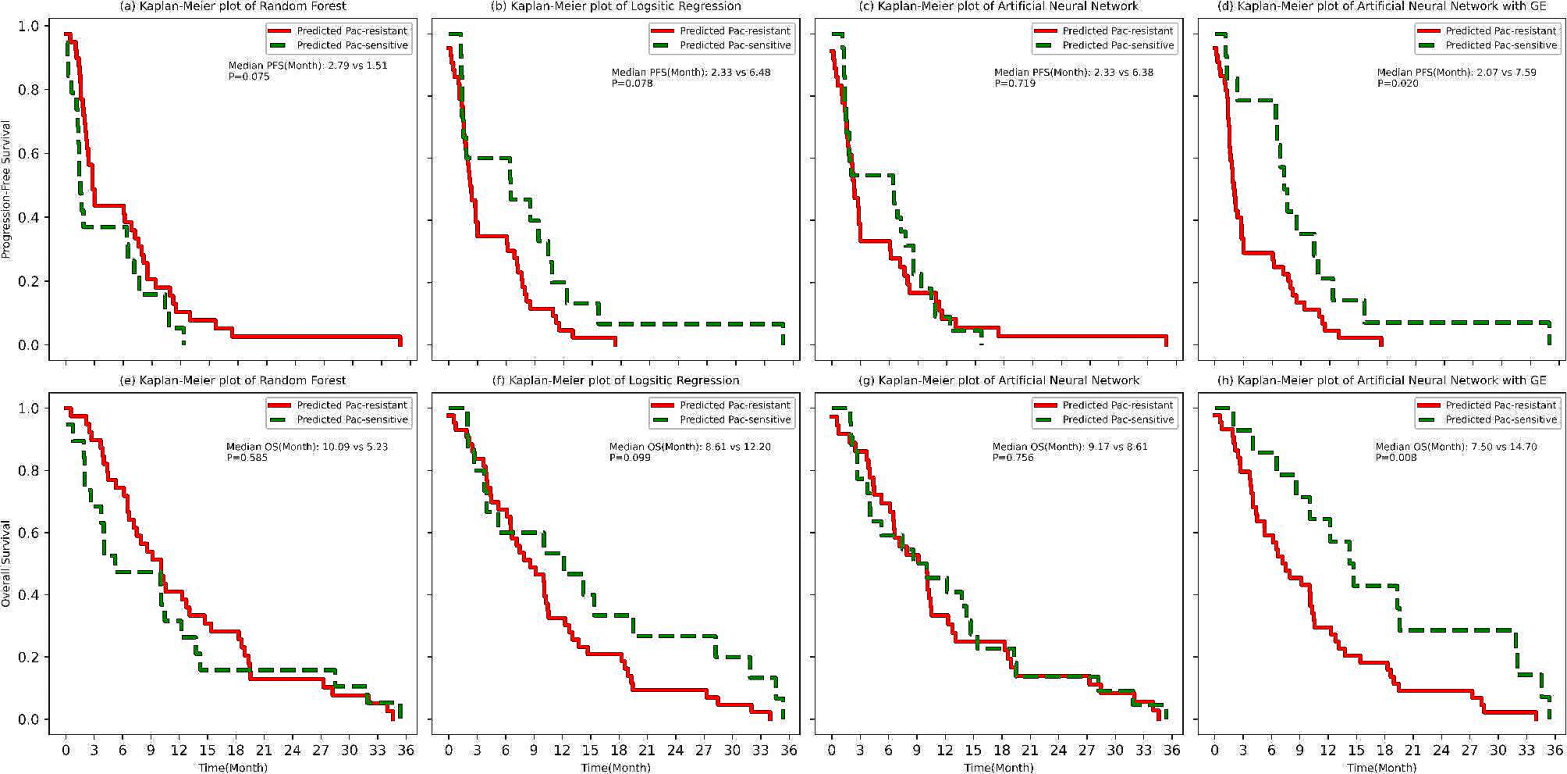
Kaplan–Meier curves of progression-free survival and overall survival in validation sets according to machine-learning methods

In the validation sets, the RF model was unable to effectively predict a longer PFS for paclitaxel-sensitive patients compared to paclitaxel-resistant ones, with median PFS figures of 1.51 vs. 2.79 months, respectively (*P* = 0.075) (Fig. 4a). Conversely, the LR model suggested a trend towards longer PFS for paclitaxel-sensitive patients (median PFS 6.48 vs. 2.33 months, *P* = 0.078) (Fig. 4b), while the ANN model indicated a non-significant numerical advantage in PFS for paclitaxel-sensitive patients over paclitaxel-resistant patients (median PFS 6.38 vs. 2.33 months, *P* = 0.719) (Fig. 4c). The ANN with GE model was the only one to significantly predict longer PFS for paclitaxel-sensitive patients (median PFS 7.59 vs. 2.07 months, *P* = 0.020) (Fig. 4d).

Regarding OS, no significant differences were noted between paclitaxel-sensitive and paclitaxel-resistant patients in both the RF and ANN models (Fig. 4e and g). The LR model, however, showed a trend towards longer OS for paclitaxel-sensitive patients (median OS 12.20 vs. 8.61 months, *P* = 0.099) (Fig. 4f). Consistently, the ANN with GE model predicted a significant extension in OS for paclitaxel-sensitive patients compared to their resistant counterparts (median OS 14.70 vs. 7.50 months, *P* = 0.008) (Fig. 4h).

## Discussion

This study showed that integrated clinical and genomic models could predict which patients with AGC are more likely to benefit from second-line paclitaxel-based chemotherapy. Among the four ML-based models, the best model was the ANN with GE model, which significantly predicted paclitaxel-sensitive or paclitaxel-resistant patients with AGC. Our ANN with the GE model aggregated the embedded genetic variants with clinical features, followed by a feed-forward neural network. The main strength of this study was that the results arose from a prospectively collected database of the K-MASTER project, a nationwide program that has maintained high-quality genomic profiling, and the relatively large size of comprehensive datasets, including both clinical and NGS data [23].

Clinical decisions to proceed with further treatment and to choose the optimal chemotherapy regimen are always challenging. Most patients with AGC become more fragile, especially after the failure of first-line chemotherapy. Additionally, chemotherapy occasionally worsens the clinical condition owing to toxicity without providing benefits. Clinical deterioration with ineffective chemotherapy may lead to a loss of chance for subsequent treatment. Currently, there are no predictive models or systems capable of determining the potential benefits of palliative chemotherapy for patients with cancer or identifying the most effective chemotherapy regimens. In the era of AI, ML-based models can be used as clinical decision-support systems [26]. Owing to technological advances in genomic profiling, NGS testing is a routine workup in oncology, and the incorporation of complicated NGS results into clinical decisions is important for individualized therapy. In addition to genomic data, numerous clinical factors must be considered when making clinical decisions. It can easily organize and interpret data from clinical practice. Thus, our ML models can serve as important backbones for future clinical decision support systems.

It is interesting to note that recently, using ML methods, the first and largest performed study identified a gene signature predictive of paclitaxel benefit in GC from the phase 3 SAMIT trial evaluating adjuvant chemotherapy [18]. A custom-designed NanoString panel, including genes involved in chromosomal stability or immunogenic cell death, was used, and an ML model identified a gene signature for predicting paclitaxel benefit [18]. Similarly, our study predicted the benefits of paclitaxel in patients with AGC using ML methods. However, an important point of consideration is that our study focused on palliative settings, which use paclitaxel as standard chemotherapy, and more caution is warranted to balance the benefits and toxicity in vulnerable patients. Our ML models integrated clinical factors and genomic data, where NGS tests can be routinely performed rather than gene signature analyses in real-world practice.

Previously, prognostic factor analysis using pooled data from two pivotal phase 3 trials evaluating second-line ramucirumab alone or ramucirumab + paclitaxel chemotherapy found 12 independent factors for poor survival, including several clinical and laboratory findings [27]. Another retrospective study similarly reported the prognostic significance of clinicolaboratory factors associated with second-line chemotherapy efficacy [12]. Good performance status and a long duration of prior first-line chemotherapy are commonly associated with better survival. However, genomic data reflecting the underlying tumor biology were not analyzed in either study. Although our study did not evaluate laboratory findings, genomic alterations were comprehensively and in-depth considered when developing the prediction models. Future research is required to expand and update our models based on various factors, including laboratory findings.

This study had few limitations. First, as a retrospectively designed study using an already established dataset, additional factors could not be analyzed. Second, there may be selection bias between the training and validation sets, despite being randomly assigned and having no statistically significant differences between them. Third, although internal validation was performed, as the small size of the validation set may not guarantee the generalizability of models, our ML models must be validated using an external independent dataset. Fourth, a prospective clinical trial is required to confirm the clinical utility of these prediction models. Finally, ML models should be incorporated into real-world practices and advanced independently if true AI-based models are used.

## Conclusions

Our ML models integrated clinical and genomic factors and identified patients with AGC with a greater likelihood of benefit from second-line paclitaxel chemotherapy. This study provides the foundation for future advanced prediction ML models.

### Electronic supplementary material

Below is the link to the electronic supplementary material.

Supplementary Material 1

## Data Availability

The datasets generated and/or analyzed during the current study are available from the corresponding author (JHK) upon request. The data are not publicly available because they contain information that can compromise the participants’ privacy/consent.

## Abbreviations

AGC: advanced gastric cancer
AUROC: area under the receiver operating characteristic curve
ML: machine learning
RF: random forest
LR: logistic regression
ANN: artificial neural network
ANN: artificial neural network with genetic embedding
HER2: human epidermal growth factor receptor 2
PFS: progression-free survival
OS: overall survival
NGS: next-generation sequencing
CNV: copy-number variation
SNV: single nucleotide variant
