# Supplementary material for: Integrated clinical and genomic models using machine-learning methods to predict the efficacy of paclitaxel-based chemotherapy in patients with advanced gastric cancer
Source: BMC Cancer. 2024 Apr 20;24:502. doi: 10.1186/s12885-024-12268-9 (PMC11031899; doi:10.1186/s12885-024-12268-9)
Supplement: Supplementary file 1 — Supplementary Material 1 [file 12885_2024_12268_MOESM1_ESM.docx]

Additional file 1.

Genes: ALK, APC, AR, ARID1A, ASNS, ASXL1, ATM, AXIN2, BARD1, BCOR, BRAF, BRCA1, BRCA2, CALR, CCND1, CCNE1, CDH1, CDK12, CDK4, CDKN2A, CEBPA, CSF3R, CTNNB1, DNMT3A, EGFR, ERBB2, ERBB3, ERBB4, ERCC2, ESR1, EZH2, FANCA, FBXW7, FGF3, FGFR1, FGFR2, FGFR3, FLT3, GNA11, GNAQ, GNAS, HRAS, IDH2, KIT, KMT2D, KRAS, MAP2K1, MAPK1, MDM2, MET, MLH1, MRE11, MSH2, MSH6, MTOR, MYC, NCOA3, NF1, NKX2-1, NOTCH1, NRAS, PBRM1, PIK3CA, PIK3R1, POLE, PRDM1, PTCH1, PTEN, PTPN11, PTPRD, RB1, RICTOR, RIT1, RUNX1, SDHB, SF3B1, SMAD4, SMARCA4, SMO, SRSF2, STAT3, STK11, TERT, TET2, TOP1, TP53, WT1

Copy Number Variations: APC_CNV, ASNS_CNV, CALR_CNV, CCND1_CNV, CCNE1_CNV, CDK4_CNV, CEBPA_CNV, EGFR_CNV, ERBB2_CNV, FGF3_CNV, FGFR2_CNV, KIT_CNV, KRAS_CNV, MAPK1_CNV, MDM2_CNV, MET_CNV, MTOR_CNV, MYC_CNV, NCOA3_CNV, PIK3CA_CNV, PRDM1_CNV, PTEN_CNV, RICTOR_CNV, RUNX1_CNV, SMAD4_CNV, SMARCA4_CNV, SRSF2_CNV, TERT_CNV, TOP1_CNV

Single Nucleotide Variants: ALK_SNV, APC_SNV, ARID1A_SNV, AR_SNV, ASXL1_SNV, ATM_SNV, AXIN2_SNV, BARD1_SNV, BCOR_SNV, BRAF_SNV, BRCA1_SNV, BRCA2_SNV, CALR_SNV, CDH1_SNV, CDK12_SNV, CDKN2A_SNV, CSF3R_SNV, CTNNB1_SNV, DNMT3A_SNV, EGFR_SNV, ERBB2_SNV, ERBB3_SNV, ERBB4_SNV, ERCC2_SNV, ESR1_SNV, EZH2_SNV, FANCA_SNV, FBXW7_SNV, FGFR1_SNV, FGFR2_SNV, FGFR3_SNV, FLT3_SNV, GNA11_SNV, GNAQ_SNV, GNAS_SNV, HRAS_SNV, IDH2_SNV, KIT_SNV, KMT2D_SNV, KRAS_SNV, MAP2K1_SNV, MET_SNV, MLH1_SNV, MRE11_SNV, MSH2_SNV, MSH6_SNV, MTOR_SNV, NF1_SNV, NKX2-1_SNV, NOTCH1_SNV, NRAS_SNV, PBRM1_SNV, PIK3CA_SNV, PIK3R1_SNV, POLE_SNV, PRDM1_SNV, PTCH1_SNV, PTEN_SNV, PTPN11_SNV, PTPRD_SNV, RB1_SNV, RIT1_SNV, RUNX1_SNV, SDHB_SNV, SF3B1_SNV, SMAD4_SNV, SMARCA4_SNV, SMO_SNV, STAT3_SNV, STK11_SNV, TET2_SNV, TP53_SNV, WT1_SNV
